# Outcomes of Renal Trauma in Indian Urban Tertiary Healthcare Centres: A Multicentre Cohort Study

**DOI:** 10.1007/s00268-021-06293-z

**Published:** 2021-08-21

**Authors:** Bhakti Sarang, Nakul Raykar, Anita Gadgil, Gunjan Mishra, Martin Gerdin Wärnberg, Amulya Rattan, Monty Khajanchi, Kapil Dev Soni, Monali Mohan, Naveen Sharma, Vineet Kumar, Deepa KV, Nobhojit Roy, On behalf of the Towards Improved Trauma Care Outcomes TITCO-India

**Affiliations:** 1 Department of Surgery, Terna Medical College and Hospital, New Mumbai, India; 2 WHO Collaborating Centre for Research in Surgical Care Delivery in LMIC, Mumbai, India; 3 Trauma and Emergency Surgery, Brigham and Women’s Hospital, Boston, MA USA; 4 Program in Global Surgery and Social Change, Harvard Medical School, Boston, MA USA; 5 Department of Surgery, Bhabha Atomic Research Centre Hospital, Mumbai, India; 6 Department of Surgery, Mahatma Gandhi Mission Medical College and Hospital, New Mumbai, India; 7 Department of Global Public Health, Karolinska Institutet, Stockholm, Sweden; 8 Function Perioperative Medicine and Intensive Care, Karolinska University Hospital, Solna, Sweden; 9 Department of Trauma Surgery and Critical Care, All India Institute of Medical Sciences, Rishikesh, India; 10 Department of Surgery, Seth GS Medical College and KEM Hospital, Mumbai, India; 11 Critical and Intensive Care, JPN Apex Trauma Hospital, AIIMS, New Delhi, India; 12 Health Systems Strengthening, Muzaffarpur Field Health Laboratory, CARE-India, Patna, Bihar India; 13 Department of General Surgery, All India Institute of Medical Sciences, Jodhpur, India; 14 Department of Surgery, Lokmanya Tilak Municipal Medical College and Hospital, Mumbai, India; 15 Department of Surgery, Manipal Hospital, Dwarka, Delhi, India; 16 Department of Global Public Health, Karolinska Institutet, 171 77 Stockholm, Sweden

## Abstract

**Background:**

Renal trauma is present in 0.5–5% of patients admitted for trauma. Advancements in radiologic imaging and minimal-invasive techniques have led to decreased need for surgical intervention. We used a large trauma cohort to characterise renal trauma patients, their management and outcomes.

**Methods:**

We analysed “Towards Improved Trauma Care Outcomes in India” cohort from four urban tertiary public hospitals in India between 1st September 2013 and 31st December 2015. The data of patients with renal trauma were extracted using International Classification of Diseases 10 codes and analysed for demographic and clinical details.

**Results:**

A total of 16,047 trauma patients were included in this cohort. Abdominal trauma comprised 1119 (7%) cases, of which 144 (13%) had renal trauma. Renal trauma was present in 1% of all the patients admitted for trauma. The mean age was 28 years (SD-14.7). A total of 119 (83%) patients were male. Majority (93%) were due to blunt injuries. Road traffic injuries were the most common mechanism (53%) followed by falls (29%). Most renal injuries (89%) were associated with other organ injuries. Seven of the 144 (5%) patients required nephrectomy. Three patients had grade V trauma; all underwent nephrectomy. The 30-day in-hospital mortality, in patients with renal trauma, was 17% (24/144).

**Conclusion:**

Most renal trauma patients were managed nonoperatively. 89% of patients with renal trauma had concomitant injuries. The renal trauma profile from this large cohort may be generalisable to urban contexts in India and other low- and middle-income countries.

## Introduction

Injuries account for 10% of global mortality which translates to around 4.5 million annual deaths [1]. It is estimated that more than 1000 non-fatal injuries occur for every injury related death [2, 3]. Globally, renal trauma is present in approximately 0.5–5% of patients with traumatic injury and 10–20% of patients with abdominal trauma [4–6].

The American Association for the Surgery of Trauma (AAST) classifies renal trauma into five grades of increasing severity [7]. Advances in imaging and endovascular techniques in high-income settings (HICs) have resulted in an increase in nonoperative management of renal trauma, including those with high AAST grades (III–V) [8–10]. Nephrectomy in these settings is often reserved for patients with persistent shock or sepsis, or when endovascular techniques or intensive care facilities are limited [10, 11].

Several recent studies from India and other low- and middle-income countries (LMICs) have also described renal trauma management with a nonoperative approach but these are all single centre studies with small sample sizes [12–14]. We used a large trauma cohort encompassing four public urban tertiary healthcare centres in India [15] to characterise patients with renal trauma and observe their management and outcomes.

## Methods

### Study design

We analysed the prospective multicentre cohort “Towards Improved Trauma Care Outcomes (TITCO) in India” [15] cohort from four urban tertiary public hospitals in India between 1st September 2013 and 31st December 2015.

### Study setting

The four hospitals included in the TITCO cohort provide Level 1 trauma services to an urban Indian population. Level 1 trauma care facilities in India provide the highest level of definitive and comprehensive coverage of all surgical specialities round the clock [16]. These hospitals in metropolitan cities (Mumbai-2; Delhi-1; Kolkata-1) received direct admissions as well as referrals from other hospitals. The participating hospitals were Jai Prakash Narayan Apex Trauma Centre of All India Institute of Medical Sciences (AIIMS), New Delhi; Lokmanya Tilak Municipal General Hospital (LTMGH) and King Edward Memorial (KEM) Hospital, Mumbai and Seth Sukhlal Karnani Memorial Hospital (SSKM), Kolkata. All sites had expertise and availability for endourology and interventional radiology.

All the study sites had availability of endourology and endovascular modalities like angioembolisation; however, the details of its utility for management of renal trauma have not been mentioned or captured in the data registry. Also, although these modalities were available at these hospitals during the study period, their utility was reserved mainly for elective procedures and not readily available as a part of emergency care of trauma patients.

### Eligibility criteria

The TITCO cohort included all trauma patients who presented to the emergency departments (EDs) of the study hospitals and were admitted for further treatment. Patients who died after arrival but before admission were also included. Patients with isolated limb injury or who were dead on arrival were not included. Patients were followed up for 30 days from the date of admission or until death or discharge from hospital, whichever was longer. The data of patients with renal trauma with or without other organ injuries, from this cohort, were extracted using the International Classification of Diseases 10 codes (ICD-10 Version:2010) [17], ICD code S37.0 specific for renal trauma.

### Data collection

Data were collected prospectively by research officers with a postgraduate degree in health sciences. The research officers received training before the data collection and guidance from the investigators on a weekly basis during the period of data collection. They worked daily through an 8-h shift in rotations with morning, evening and night duties. They used a standardised intake form for data collection and made entries by directly observing the doctors and paramedical staff, engaged in trauma care during their respective duty hours. The data for the patients admitted outside the duty hours were retrieved from patient’s medical records the subsequent day. They uploaded the data to a central database weekly and the team of investigators from each centre checked the data periodically to ensure quality and consistency of data elements. The Injury Severity Score (ISS) was calculated based on the details of injury, imaging and operative findings by a certified abbreviated injury scale specialist.

### Study variables

We analysed the data of patients with renal trauma for the characteristics including age and gender, mechanism of injury, heart rate (HR) and systolic blood pressure (SBP), Glasgow Coma Score (GCS) and Injury Severity Score (ISS). The data also included information on haemoglobin levels, blood urea nitrogen levels, contrast enhanced computerised tomography (CECT) findings, surgical interventions if any, length of hospital stay and blood transfusion details.

We categorised each renal injury into AAST grades based on the CECT reports and/or operative findings. The renal injuries whose description was not detailed in the CECT reports or operative notes were not assigned any grade and marked as non-gradable (NG).

All the collaborating centres approved the TITCO study in their respective institutional review board, and a waiver of informed consent was granted. This study used anonymised data from the TITCO cohort.

### Quantitative variables and statistical methods

All continuous variables were represented as median and interquartile range and categorical variables as counts and percentages. Age was represented as mean with standard deviation. The data analysis was performed using Microsoft Excel statistical software 2019.

## Result

**Fig. 1:**
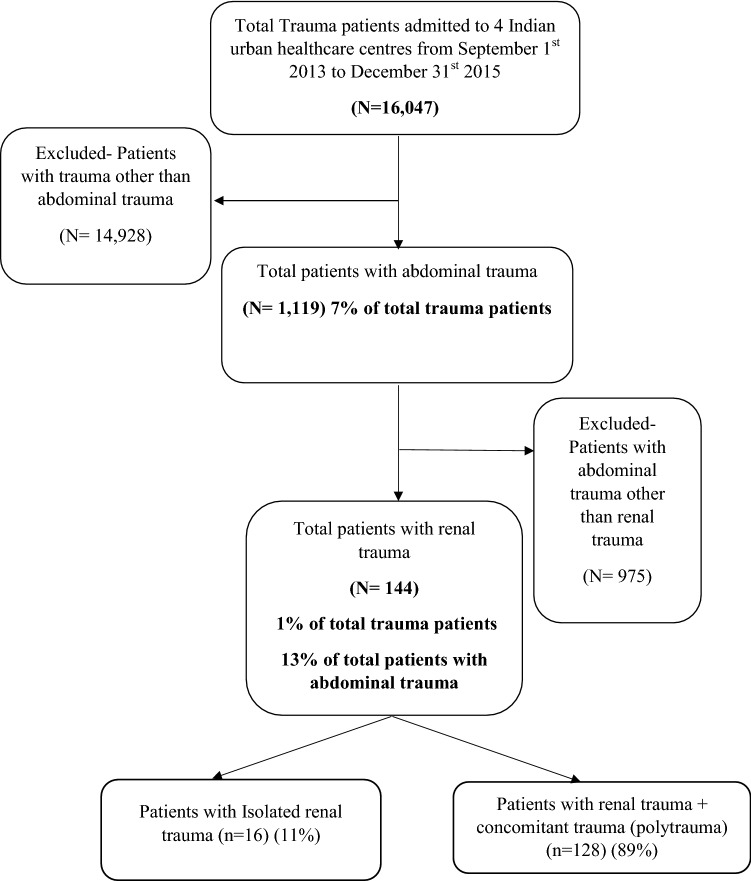
Recruitment algorithm

Over the study period, 16,047 trauma patients were admitted and enrolled in the TITCO cohort. Abdominal trauma comprised 1119 (7%) cases, of which 144 (13%) had renal trauma. Renal trauma was present in 1% of patients admitted for trauma (Fig. 1).

**Fig. 2:**
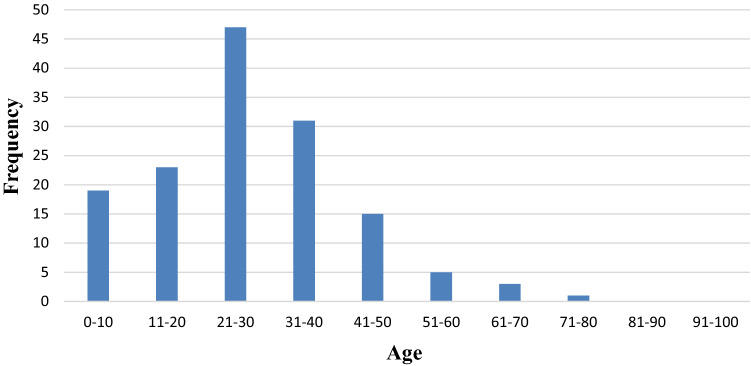
Age distribution in patients with renal trauma

The demographic and clinical profile of the patients with renal trauma listed is shown in Table 1. The mean age was 28 years (SD-14.7), age following a unimodal distribution, with young adults (20–40 years), being the most affected (Fig. 2). A total of 119 (83%) patients were male. Blunt trauma was the predominant mode of injury (93%). Road traffic injuries (RTIs) were the commonest mechanism (53%) followed by falls (29%). On sub-categorisation of patients with renal trauma due to RTIs, we observed that pedestrians and motorcyclists constituted around 47% (36/76) of patients having renal trauma. Grade V renal trauma was seen in motorcyclists and drivers. 76% of patients with renal trauma had no or mild traumatic brain injury (TBI) with GCS 13–15. 3% and 14% patients had moderate (GCS 9–12) and severe (GCS ≤ 8) TBI, respectively. The median ISS was 17 (IQR- 9–21). Patients with grade IV and V renal trauma had predominantly severe (16–25) and profound (26–75) ISS. However, the ISS in these patients did not correlate with the need for surgical intervention. A total of 24 (17%) patients with renal trauma were hypotensive (SBP < 90 mm Hg) on presentation and 50 (35%) had tachycardia (HR > 100 beats/minute) (Table 1).

**Table 1:** Demographics and Clinical Profile of Patients with Renal Trauma

Variables	Value *N* = 144 *N* (%)	Missing values (*n*)
Age	28 (14.7)	0
Males	119 (83)	0
Adults (> 18 years of age)	115 (80)	0
Mechanism of injury (MOI) Road traffic injury Falls Assault Railway injuries Others	76 (53) 41 (29) 14 (10) 5 (4) 8 (6)	0
Blunt injury	134 (93)	0
Isolated renal trauma Renal trauma + Concomitant trauma	16 (11) 128 (89)	0
AAST grade I II III IV V NG	14 (10) 45 (31) 37 (26) 27 (19) 3 (2) 18 (13)	
HR	90 (80–109)	2
Tachycardia (HR > 100 bpm)	50 (35)	
SBP	115 (102–124)	4
Hypotension (SBP < 90 mmHg)	24 (17)	
GCS Severe TBI (≤ 8) Moderate TBI (9–12) Mild TBI (13–15)	20 (14) 5 (3) 109 (76)	10
ISS	17 (9–21)	34
Haemoglobin (gm/dl)	11.6 (9.6–13.2)	10
Blood transfusion in the first 24 h	39 (27)	
Blood urea nitrogen (mg/dl)	27 (20.3–35.5)	11
Length of stay (days)	6 (4–15)	1

Continuous variables are represented by median and interquartile range

Categorical variables are represented as counts and percentages

Age represented as mean and standard deviation, TBI- Traumatic Brain Injury

All percentages rounded up to the closest integer, NG- Non-Gradable based on CECT or operative findings

**Fig. 3:**
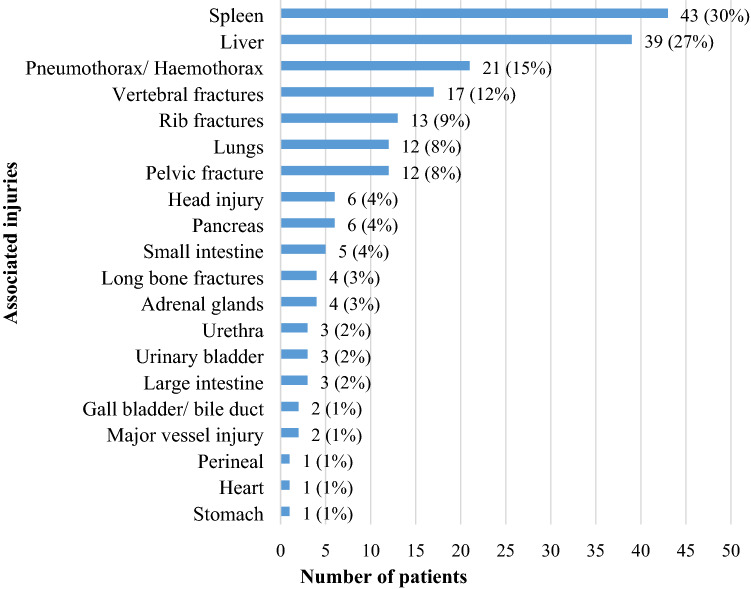
Organ injuries associated with renal trauma

Most patients with renal trauma (89%) had other concomitant organ injuries. Only 16 (11%) patients had isolated renal trauma. Splenic (30%) and liver (27%) injuries were most associated with renal trauma (Fig. 3).

Operative management (OM) for the kidney was performed in 10 patients. Seven of the 144 (5%) patients had nephrectomy. Of the seven patients who underwent nephrectomy, five were operated within the first 24 h of admission while the remaining two had delayed nephrectomy (beyond 24 h, exact timing unknown). Three patients underwent primary repair of renal injury (renorrhaphy).

Three patients had grade V trauma; all underwent nephrectomy due to haemodynamic instability. One fourth of patients with grade IV trauma (*n* = 7) underwent OM; 4 patients underwent nephrectomy, none due to haemodynamic instability; 3 had primary repair of the renal injury (Table 2).

**Table 2:** Management of renal trauma based on the AAST grades

Grade	NOM	OM (Renal repair/associated injuries)	OM Nephrectomy	Nephrectomy rate	Isolated renal trauma	Mortality
I (*n* = 14)	12	0/2	0	0	0	3 (21%)
II (*n*( = 45)	33	0/12	0	0	2	9 (20%)
III (*n* = 37)	28	0/9	0	0	9	3 (8%)
IV (*n* = 27)	16	3/6*	4	15%	4	4 (15%)
V (*n* = 3)	0	0/0	3	100%	0	1 (33%)
NG (*n* = 18)	13	0/5	0	0	1	4 (22%)

*2 of the 3 patients undergoing OM for renal repair were also operated for concomitant splenectomy. Hence 3/6, wherein 2 patients are common on both sides

OM- Operative Management, NOM- Nonoperative Management, NG- Non-Gradable

The 30-day in-hospital mortality in patients with renal trauma was 17% (24/144). The mortality in patients with renal trauma as per AAST grades is shown in Table 2. However, due to overall small number of patients with renal trauma with very few patients having isolated renal trauma, its contribution to the mortality could not be determined.

## Discussion

We found renal trauma to be present in around 1% of patients admitted for trauma and 13% of patients with abdominal trauma in this Indian cohort. This is comparable to global trauma literature with renal trauma present in around 0.5–5% of patients admitted for trauma and 8–20% of patients with abdominal trauma [4, 6, 7, 18].

Most patients were managed conservatively (NOM) with only 7 (5%) undergoing nephrectomy. Nephrectomies were only performed in patients with grade IV & V renal trauma. Indian centres have previously reported nephrectomy rates of 12–16%, with those in grade V approaching 90% [12, 19]. A study from South Africa, a setting similar to the Indian LMIC context, demonstrated high nephrectomy rates of 40% for grade IV and 89% for grade V in predominantly blunt renal trauma [20]. Contrary to this, another South African study from a tertiary level major trauma centre utilising endovascular and endourological interventions for trauma management, demonstrated nephrectomy rates of 10.5% for grade IV and 25% for grade V patients with blunt renal trauma [21]. A genitourinary trauma study by the AAST demonstrated nephrectomy rates of 15% for grade IV and 62% for grade V [10], and a Canadian study by Mann et al. demonstrated only a 4% nephrectomy rate for high-grade renal trauma [22]. As compared to HICs and LMIC setups practicing minimally invasive endovascular and endourological techniques, the nephrectomy rate was higher in our cohort for grade V renal trauma. However, a true comparison may not be feasible due to the small number of patients with grade V renal trauma in our cohort and all of these patients being hemodynamically unstable, at least upon arrival. Further, while angioembolisation was available in all the centres included in this study, utilisation of these resources is not recorded in our dataset. Additionally, access to these resources in the emergency trauma setting can be variable based on time of day and existing case volume.

Renal trauma often occurs as a part of polytrauma. 89% of renal trauma patients in this study had concomitant injuries. The most common organs affected were the spleen and the liver. This is comparable to the literature around the world with 80–95% patients having associated organ injuries [7, 23, 24]. We observed a relatively high mortality in patients with low-grade renal trauma and other concomitant injuries. This high mortality was likely due to serious concomitant injuries, as isolated renal trauma in our cohort was rare.

The mean age of patients afflicted with renal trauma in this study was 28 years. Age demonstrated a unimodal distribution with young adults being the most affected. We also observed a male predominance. Blunt injuries, particularly RTIs and falls, were the most common mechanism . This is similar to the global literature, wherein patients afflicted with renal trauma have been predominantly young, with a mean age between 30 and 40; mostly male, reflecting 70–90% of cases; and due to blunt trauma mechanism, particularly road traffic injury [7, 23, 25]. Renal trauma was observed mainly in pedestrians and motorcyclists among the RTIs. Pedestrians and motorcyclists are vulnerable to renal trauma, and these injuries may be unique to LMIC settings like India [10] and may form basis for future work.

A strength of this analysis is the large dataset, representing multiple level 1 public urban healthcare centres across India, allowing a more complete representation of the renal trauma patient profile, injury grade, and management strategies employed.

A limitation of our analysis is the limited information on the use of endovascular therapies. Additional research is needed to understand the impact of the availability of endovascular therapy, or its absence, on the management of high-grade renal trauma in the Indian context.

## Conclusion

Renal trauma is present in 1% of patients admitted for trauma and 13% of patients with abdominal trauma. Most renal trauma patients were managed nonoperatively. Most patients (89%) with renal trauma had concomitant injuries. The renal trauma profile from this large cohort may be generalisable to other urban healthcare centres in India and urban contexts in other LMICs.
